# COVID-19 and the Secular Theodicy: On Social Distancing, the Death of God and the Book of Job

**DOI:** 10.1007/978-3-030-65355-2_7

**Published:** 2021-03-20

**Authors:** Frank Bosman, Archibald van Wieringen

**Affiliations:** 1 Tilburg University, Tilburg, The Netherlands; 2 Tilburg University, Tilburg, The Netherlands; 3 Tilburg University, Tilburg, The Netherlands; 4 Tilburg University, Tilburg, The Netherlands; 5 Department of Systematic Theology and Philosophy, Tilburg School of Catholic Theology, Tilburg, The Netherlands; 6 Department of Biblical Sciences and Church History, Tilburg School of Catholic Theology, Tilburg, The Netherlands

## Abstract

In times of great distress, like in the case of the COVID-19 pandemic, people look for relief from the existential threat by searching for some kind of interpretation of the crisis. Some people will look for scapegoats to put the blame on, while others will search for ways by which the crisis can also be perceived as something beneficial.

As far as the COVID-19 pandemic goes, earlier this year, media and politicians pointed towards China, where the pandemic started, or to Italy, from where the virus spread over the European continent.

Since the beginning of the crisis, we have also been flooded with gurus, motivational speakers, and mindfulness coaches who stimulate us to view the new common as an unexpected but much needed “reboot” of our day-to-day life.

Intriguingly enough, these two individual and collective coping strategies are very familiar to those who are acquainted with the Christian philosophical and theological traditions. When confronted with the apparent paradox between the idea of an omnipotent and omnibenevolent deity on the one hand and the experience of everyday pain and suffering on the other hand, Christians have sought for ways to find a satisfactory solution. This is known as theodicy. As the Roman and Christian philosopher Boethius summarized the problem: si Deus, unde malum? “If God exists, wherefrom evil?”

In times of great distress, like in the case of the COVID-19 pandemic, people look for relief from the existential threat by searching for some kind of interpretation of the crisis. Some people will look for scapegoats to put the blame on, while others will search for ways by which the crisis can also be perceived as something beneficial.

As far as the COVID-19 pandemic goes, earlier this year, media and politicians pointed towards China, where the pandemic started, or to Italy, from where the virus spread over the European continent. The Chinese and the Italians became COVID-19’s scapegoats. Others even re-kindled old conspiracy theories involving Freemasons, the Vatican, and the CIA.

Since the beginning of the crisis, we have also been flooded with gurus, motivational speakers, and mindfulness coaches who stimulate us to view the new common as an unexpected but much needed “reboot” of our day-to-day life, an escape from the rat race of postmodern life, or as a spiritual detox of our material existence (Bezemer 2020; Cappelle 2020; Mascini 2020; van Raaij 2020).

## Classical Theodicy

Intriguingly enough, these two individuals and collective coping strategies are very familiar to those who are acquainted with the Christian philosophical and theological traditions. When confronted with the apparent paradox between the idea of an omnipotent and omnibenevolent deity on the one hand and the experience of everyday pain and suffering, on the other hand, Christians have sought for ways to find a satisfactory solution. This is known as theodicy. As the Roman and Christian philosopher Boethius summarized the problem: si Deus, unde malum? “If God exists, wherefrom evil?”

In monotheistic religions, the problem of evil was contextualized differently. In religions with two or more godheads, suffering could easily be attributed to one divine source while the good could be attributed to the other one.

In dualistic circles, such as those of the Manichean and the Cathars, the reality could be divided into a “good principle” and a “bad” one. In doing so, the “good” could be held separately from the “bad.” In monotheist thinking, this option is impossible.

In later Christian philosophy and theology, the two most important schools of thought were the Irenaean and the Augustinian theodicies, named after Irenaeus of Lyon (c. 130–202) and Augustine of Hippo (354–430) (Hick 1996). The Irenaean solution is to “excuse” evil and suffering by relating them to a higher and better goal. Suffering is indeed caused by God but for the benefit of humankind: to learn a truth, to grow spiritually, or to prove the persistence of one’s faith. The Augustinian approach is to excuse God, redirecting the cause of suffering to humanity’s free will. God will not stop human suffering, because it is our responsibility to prevent it and to cope with its consequences.

## Secular Theodicy

In our times, after the well-known proclamation of the “death of God” by Friedrich Nietzsche and many others, the question of the theodicy has, perhaps strangely at first sight, not vanished although it has been modified to fit within an atheist, secular framework (Bosman 2019: 125–149). The question now is si non Deus, unde malum? “If God does not exist, wherefrom evil?” The world did not stop being a place of pain and suffering when its creator was declared obsolete. More strongly, in this chapter, we argue that the same theodicean hermeneutics can even be traced in society’s approach and reaction to the COVID-19 pandemic.

Some of us want to excuse COVID-19’s perceived evil by drawing attention to its perceived benefits, such as the aforementioned spiritual detox or escape from the rate race. The evil—the pandemic—still exists and is still causing a lot of pain, suffering, and death, but its downsides are thought to outweigh its counter-benefits. We can learn something from COVID-19, we can benefit from insights obtained from the crisis, and we get the opportunity to do things radically differently—and better—in the near future.

God, on the other hand, cannot be excused from anything since we have declared God obsolete in the secular domain of our Western European societies. This creates a new problem of its own. If there is not a supernatural, divine entity who can be blamed for a pandemic, then who is to blame? This scapegoat mechanism takes its name from a text found in the Old Testament: once a year a goat is burdened with all the sins of the people of Israel and is sent into the desert, symbolically atoning for the sins of the collective (Leviticus 16).

In our times, in the case of COVID-19, multiple scapegoats have been identified, ranging from the Chinese and the Italians, to blundering governments and singing church congregations. Stigmatizing some groups of people will not help us to overcome COVID-19. From history, we know of too many examples of how the stigmatization of groups can lead to inhumane disasters.

## Deification

From a philosophical point of view, another possible risk in our present situation is the deification of protocols, or in other words, the substitution of the old transcendental God of monotheism for an immanent uncritical belief in the human possibilities of controlling our life and containing all harm threatening it. Words like “Social Distancing Society” or the “New Normal” are not only spelled in capitals because of a specific custom found in written English but also because they testify to a new belief system that is (pre) supposed to free us of the imminent danger.

If these protocols fail—and they will, in the sense that no protocol can guarantee a hundred percent success rate—we will not blame the deified protocols themselves like we do not blame God in the Augustinian theodicy, but we will blame individual (groups of) people who are not able or not willing to obey and strictly follow all the instructions. We will not blame our new deity, but we will, once again, blame our free will.

## The Biblical Book of Job

Although the Bible does not contain the word theodicy, it does consider the question “why does evil strike innocent people?” In fact, the entire biblical Book of Job is dedicated to this question of theodicy: Job, a very righteous human being, is struck by one calamity after another—how is that possible?

Although the two theodicean hermeneutics we discussed above are present in this book, they are both rejected. Job’s friends represent the first hermeneutic focusing on evil. They try to convince Job that he should learn from this evil afflicting him, that he is a sinner. A sinner against what? The friends have no idea, but Job should realize from his being struck by the evil that he is a sinner anyway. Job refuses. This implies that the Book of Job rejects the idea advocated by all of Job’s friends. Job himself struggles with the second hermeneutic, focusing on God, and blames God for the evil he is experiencing. Not only does he hold God responsible for evil but he also wishes to hold God accountable for it. Therefore, Job wants to sue God.

However, the trial is not continued, not because Job’s protest against evil is unjustified or because God cannot be held accountable for his own responsibilities but because Job’s accusation came to the wrong shop. In the Book of Job, God does not reveal himself as the one responsible for evil, but as the one who, together with Job, is against evil and who is combating it by constructing creation amidst a chaotic environment, which is continuously prone to falling into deconstruction. And thus, the Book of Job also rejects the second hermeneutic (Schuman 2011).

## Concluding Thoughts

From the perspective of Job and of the Christian theodicy philosophies and theologies, a real theodicy answer to the COVID-19 crisis, secular or not, is neither blaming others (or the Other) nor whitewashing the pandemic by underscoring its perceived blessings in disguise. The creation of a new godhead will not save us either. Instead, the real answer is fighting the evil itself, i.e., COVID-19. The two hermeneutics, whether they are expressed in a religious or in a secular context, are counter-productive. This theological insight could benefit our society in general and our university in particular in combating the COVID-19 crisis, respecting all human dimensions.
